# Salivary and serum interleukin 1 beta, interleukin 6 and tumor necrosis factor alpha in patients with leukoplakia and oral cancer

**DOI:** 10.4317/medoral.17323

**Published:** 2011-07-15

**Authors:** Vlaho Brailo, Vanja Vucicevic-Boras, Josip Lukac, Dolores Biocina-Lukenda, Iva Zilic-Alajbeg, Aleksandar Milenovic, Melita Balija

**Affiliations:** 1 Department of Oral Medicine, School of Dental medicine, University of Zagreb, Gundulićeva 5, 10000 Zagreb, Croatia; 2 Clinic for Nuclear Medicine and Oncology, Clinical hospital “Sestre milosrdnice”, Vinogradska cesta 29, 10000 Zagreb, Croatia; 3 Study of Dentistry, Medical Faculty, University of Split, Croatia, Šoltanska 2, 21000 Split, Croatia; 4 Department of Prosthodontics, School of Dental medicine, University of Zagreb, Gundulićeva 5, 10000 Zagreb, Croatia; 5 Clinic for Maxillofacial surgery, Clinical Hospital Dubrava, Avenija Gojka Šuška 6, 10000 Zagreb, Croatia; 6 Croatian Institute for Transfusion Medicine, Petrova 3, 10000 Zagreb, Croatia

## Abstract

Objectives: The aim of study was to compare salivary and serum concentrations of interleukin 1 beta (IL-1β), interleukin 6 (IL-6) and tumor necrosis factor alpha (TNF-α) in patients with oral leukoplakia, oral cancer and healthy controls. Study design: Eighty eight patients (28 with oral cancer, 29 leukoplakia, and 31 healthy controls) were included in this study. Cytokine concentrations were measured by commercial enzyme linked immunoassay. Results: Salivary IL-1β and IL-6 were significantly higher in oral cancer patients than in patients with leukoplakia and control group (p<0.05). No differences in concentrations of salivary TNF-α between either of the groups were observed. Serum concentrations of IL-1β were below level of detection in all but two participants. No significant differences between the groups were observed in serum concentrations of IL-6. Serum TNF-α was significantly higher in control subjects than in oral cancer patients. Conclusions: Patients with oral cancer have elevated levels of inflammatory cytokines in their saliva. Whether this elevation can be used for monitoring the malignant transformation of oral leukoplakia remains to be answered by further follow up studies.

** Key words:** Cytokines, oral, leukoplakia, cancer.

## Introduction

Oral leukoplakia is defined as “white lesion of oral mucosa which can not be classified clinically or histologically as any other lesion and which is not associated with any physical or chemical causative agent except the use of tobacco”(1). Malignant transformation of oral leukoplakia occurs on average in 1% of cases per year (2). Factors associated with higher risk of malignant transformation are: female gender, occurrence of leukoplakia in non smokers, non homogenous leukoplakia and presence of epithelial dysplasia. However, malignant transformation has also been described in cases of leukoplakia without any of the previously mentioned factors (3-5). 

Proinflammatory cytokines interleukin 1 beta (IL-1β), interleukin 6 (IL-6) and tumor necrosis factor alpha (TNF-α) exert various biological functions. Apart from regulating inflammatory response, these cytokines play significant role in the development of cancer. IL-1β acts as a potent promoter of cancerogenesis by enhancing the action of chemical cancerogens which results in proliferation of mutated cells and further accumulation of genetic damage (6). 40% lower incidence of tumors in was reported in animals knocked out for IL-1β (7). IL-6 enhances secretion of transcription factor AP-2, potent cell cycle regulator that activates oncogenes Ras and c erB2 (8). Furthermore, IL-6 inactivates p53 tumor suppressor gene by supporting the hypermethylation of its’ promoter region which can result in the suppression of apoptosis and uncontrolled cell growth (9). TNF-α activates positive cell cycle regulator NF-κB which leads to evasion of apoptosis and cell proliferation (10). TNF-α can also act as potent endogenous mutagen causing direct damage to DNA through the induction of reactive oxygen species (11). Significantly higher genetic instability in cells treated with TNF-α compared to cells not treated with TNF-α was reported (11). In the same study malignant transformation of murine embryonic cells was induced by TNF-α (11). 

The aim of this study was to compare salivary and serum concentrations of IL-1β, IL-6 and TNF-α in patients with oral leukoplakia, oral cancer and healthy controls. 

## Subjects and Methods

 -Participants

Eighty eight participants were enrolled in the study which was approved by the ethical committee of the School of Dental Medicine, University of Zagreb. Prior to the enrolment, informed consent was obtained from each participant. Patients with chronic inflammatory diseases (such as arthritis, psoriasis, inflammatory bowel disease, Sjoegren’s syndrome) that might influence the levels of salivary and/or serum cytokines were not considered for the enrolment.

-Participants were divided in three groups.

First group consisted of 29 patients (16 females and 13 males) with oral leukoplakia referred to the Department of Oral Medicine. Diagnosis was confirmed by histopathologic examination according to the WHO (1). Twenty-three patients had leukoplakia without signs of dysplasia, three patients had leukoplakia with mild dysplasia and four patients had leukoplakia with moderate dysplasia. 

Second group consisted of 28 patients (6 females and 22 males) with newly diagnosed, histologically confirmed squamous cell carcinoma of the oral cavity. Size of the tumor was assessed according to the TNM classification (12). Four patients were classified as T1, six patients were classified as T2, eight patients were classified as T3 and 12 patients were classified as T4 tumor. Eleven patients had regional metastases. Distant metastases were not reported in any of the patients. 

Control group consisted of 31 participants (12 females and 19 males) with no oral mucosal pathology. 

Details about age, smoking and periodontal health are presented in ( Table 1).

-Sample collection

Saliva samples were collected between 8 and 12 A.M. Participants were asked to refrain from eating, chewing and drinking al least one hour before collection. In patients with oral carcinoma and leukoplakia salivary samples were collected before any therapeutic procedure and minimum 3 weeks after the biopsy was performed. 

Saliva was collected 5 minutes into calibrated tubes by drooling method (13). Blood was collected by vacuum collection from cubital vein. Samples were frozen and kept at -70 ˚C until analysis. After defrosting, saliva and serum samples were analyzed within 1-2 hours.

In order to assess the impact of periodontal disease on levels of salivary cytokines, Community Periodontal Index of Treatment Needs was measured in each patient after the collection of saliva (14).

Concentrations of salivary and serum IL-1β, IL-6 i TNF-α were measured by commercial chemiluminescent enzyme linked immunoassay (Immulite, Siemens, Germany).

-Statistical analysis

Normality of distribution was assessed by Smirnoff Kolmogorof’s test. Further analysis was performed by non parametric methods due to non normal distribution. Kruskal Wallis test was used for comparisons between the groups. If any differences were observed, Mann Whitney test for comparisons between two groups was performed. Values lower than 0.05 (p<0.05) were considered as statistically significant. 

## Results

Concentrations of salivary IL-1β were significantly higher in oral cancer patients than in patients with leukoplakia and control group (p≤0.05). Patients with oral leukoplakia had significantly lower concentrations of salivary IL-1β compared to control group (p≤0.05) ( Table 2).

Salivary IL-6 was significantly higher in oral cancer patients compared to patients with leukoplakia and control group (p≤0.05). No significant differences in salivary IL-6 concentrations were observed between patients with leukoplakia and control group ( Table 2). 

No differences in concentrations of salivary TNF-α were observed between the three studied groups ( Table 2).

Serum IL-1β concentrations were below the level of detection in all three groups of participants, except in one patient with oral cancer and one control participant. 

No statistically significant differences in serum IL-6 concentrations between three groups were observed ( Table 3).

Serum TNF-α concentrations in control group were significantly higher than serum TNF-α concentrations in patients with oral cancer and leukoplakia (p≤0.05) ( Table 3).

Smoking did not affect concentrations of IL-1β, IL-6 and TNF-α neither in serum, nor in saliva. No significant differences in salivary concentrations of any of the observed cytokine between smokers and non smokers were found ( Table 4).

No significant differences in serum IL-6 and TNF-α concentrations between smokers and non smokers were found ( Table 5). Serum IL-1β concentrations were below the level of detection in all but two participants.

Concentrations of salivary IL-1β, IL-6 and TNF-α were not affected by periodontal health. No significant differences in periodontal health, assessed by CPITN, were observed between the groups ( Table 1). Furthermore, no differences in salivary concentrations of any of the studied cytokines between edentulous and dentate patients with oral cancer and leukoplakia were found (data not shown) (In the control group only one patient was edentulous and analysis could not be performed).

**Table 1:**
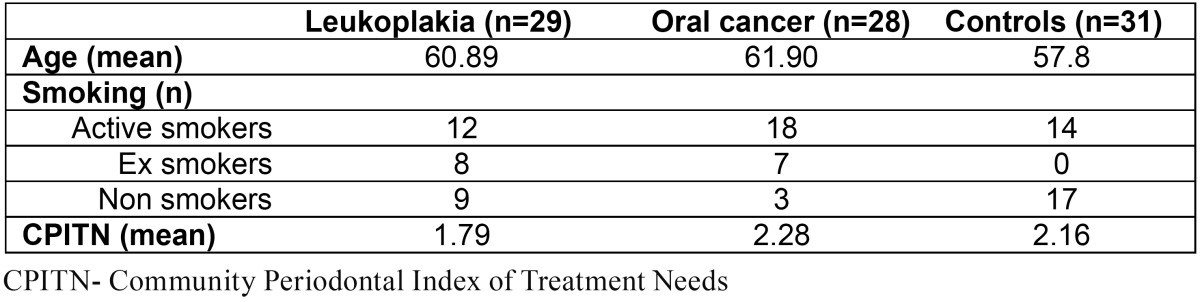
Demographic characteristics of the participants.

**Table 2:**
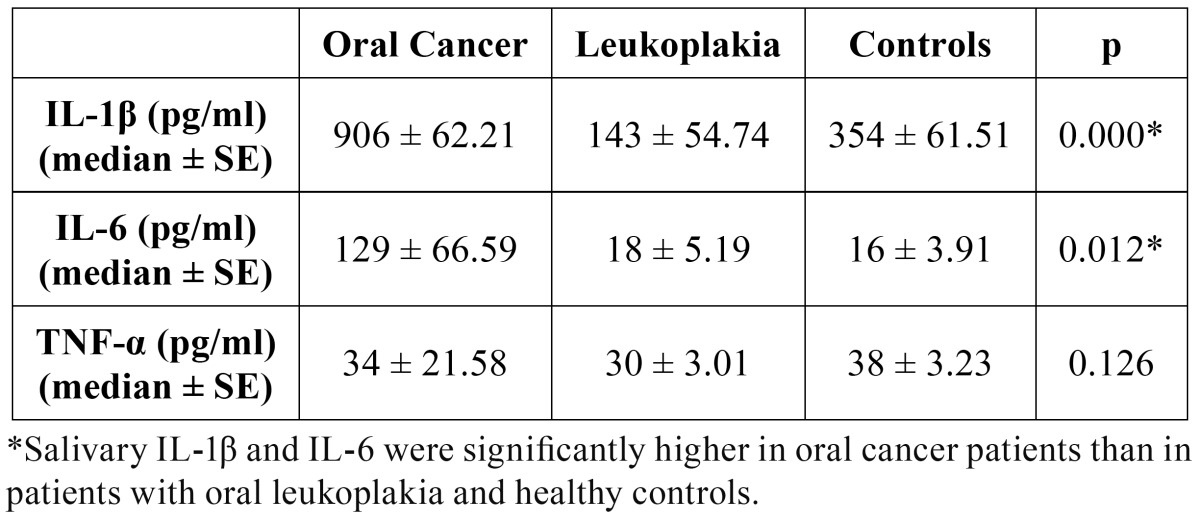
Salivary interleukin 1 beta, interleukin 6 and tumor necrosis factor alpha in patients with oral cancer, oral leukoplakia and healthy controls (pg/ml).

**Table 3:**
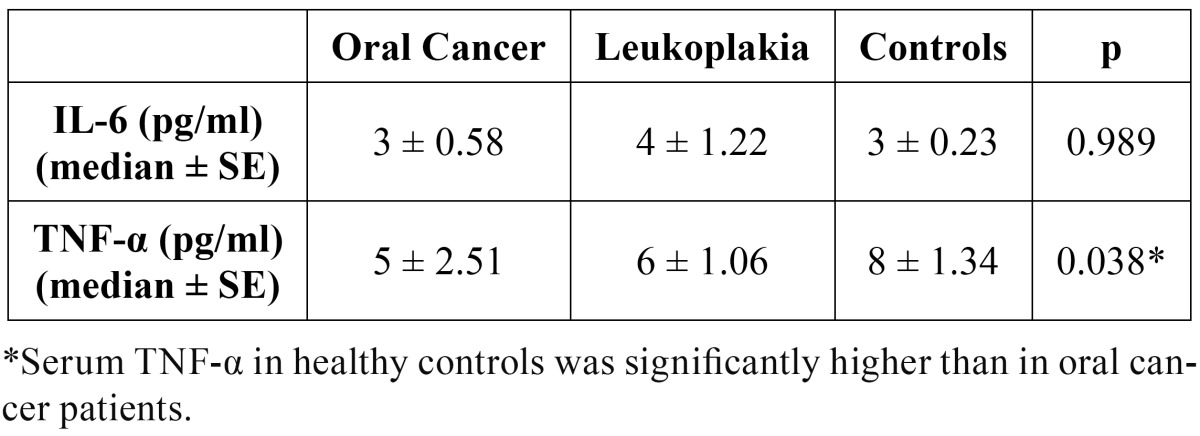
Serum interleukin 6 and tumor necrosis factor alpha in patients with oral cancer, oral leukoplakia and healthy controls (pg/ml).

**Table 4:**
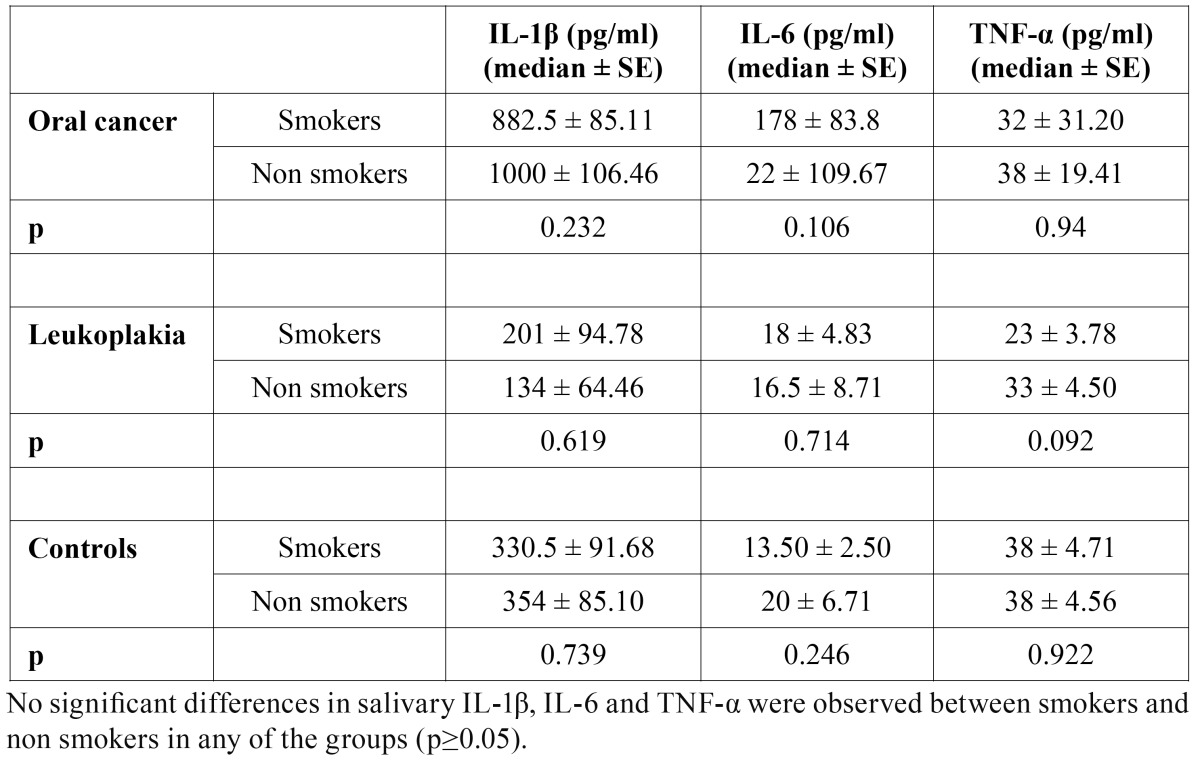
Salivary interleukin 1 beta, interleukin 6 and tumor necrosis factor alpha in smokers and non smokers (pg/ ml).

**Table 5:**
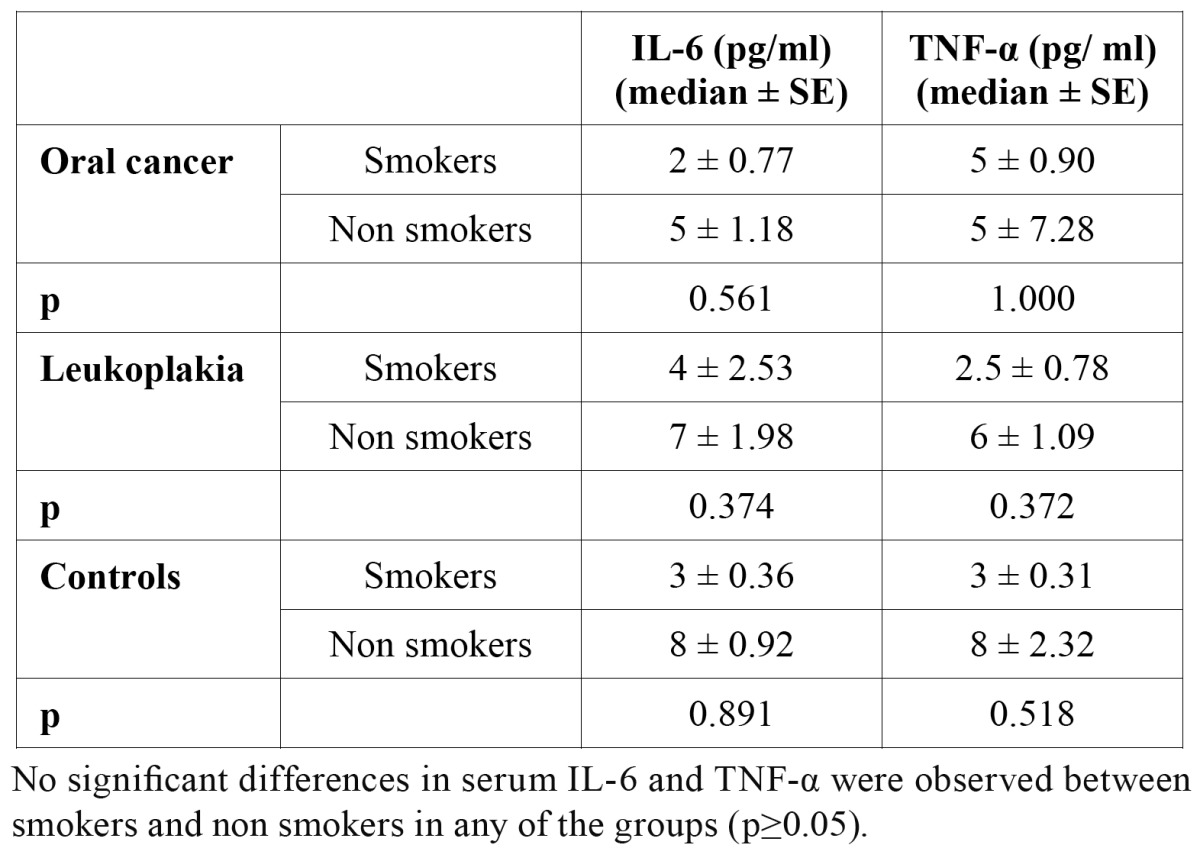
Serum interleukin 6 and tumor necrosis factor alpha in smokers and non smokers (pg/ ml).

## Discussion

Results of this study show that patients with oral cancer have higher salivary IL-1β and IL-6 concentrations compared to patients with leukoplakia and healthy controls. Increased concentrations of salivary cytokines in oral cancer patients were also reported by other authors. Arellano-Garcia et al. reported significantly higher salivary IL-1β concentrations in patients with oral cancer compared to healthy controls (15). Katakura et al. reported higher salivary IL-1β and IL-6 concentrations in oral cancer patients compared to healthy controls (16). Higher concentrations of salivary IL-6 and TNF-α in oral cancer patients and in patients with oral precancerous lesions were reported by Rhodus et al. (17,18) Saheeb Jamee et al. reported significantly higher salivary IL-6 concentrations in oral cancer patients compared to healthy controls whereas no significant differences in salivary TNF-α between the two groups could be found (19). Vučićević Boras et al. (20) reported significantly higher salivary IL-6 concentrations in oral cancer patients while St. John et al. (21) found no significant differences in salivary IL-6 in oral cancer patients compared to healthy controls. In spite of the heterogeneity of the results, one can conclude that patients with oral cancer have altered cytokine production which reflects the concentrations of at least one proinflammatory cytokine in saliva. 

Altered cytokine responsiveness is tightly associated with the development of oral cancer. While in normal cells, stimulation with proinflammatory cytokines leads to growth inhibition, in oral cancer cells stimulation with proinflammatory cytokines leads to up regulation of positive cell cycle regulators such as nuclear factor kappa B (NF-κB), signal transducer and activator of transcription (STAT) and mitogen-activated protein kinase/extracellular signal-regulated (ERK) pathway. Upregulation of these factors promotes cell survival and proliferation (22). 

Serum IL-1β concentrations were below the level of detection in all three groups which is partially in concordance with results of Wong et al. (23) who reported undetectable serum IL-1β concentrations in more than 50% of healthy individuals. Jablonska et al. (24) reported significantly higher concentrations of serum IL-1β in oral cancer patients compared to healthy controls while Hathaway et al. (25) and Hoffmann et al. (26) found no significant differences between the two groups. 

In this study no significant differences in serum IL-6 were observed between the groups. Our results are in concordance with results of Hathaway et al. (25) and Vucicevic Boras et al. (20). However, several studies reported significantly higher concentrations of serum IL-6 in oral cancer patients compared to healthy controls (21,22). Serum TNF-α concentrations were significantly higher in control subjects compared to oral cancer patients. Higher concentrations of serum TNF-α in healthy controls compared to oral cancer patients were reported by Hoffmann et al. (26) although differences did not reach statistical significance. Jablonska et al. (24) reported significantly higher concentrations of serum TNF-α in oral cancer patients compared to healthy controls while Hathaway et al. (25) found no significant differences in serum TNF-α concentrations between the two groups. 

Results from this study and heterogeneous literature data indicate that altered cytokine production and responsiveness in oral cancer takes place primarily in the oral cavity and does not reflect on serum cytokine concentrations. The most obvious example is IL-1β whose values in saliva were the highest of all studied cytokines while in serum its’ values were below the level of detection.

Our results show that patients with oral cancer have significantly higher concentrations of salivary IL-1β and IL-6 compared to patients with leukoplakia and healthy individuals. One could argue that higher concentrations of salivary cytokines might be result of a lesion with epithelial discontinuity and surrounding inflammation, not directly related to cancer. However, in the study by Rhodus et al. (18) levels of salivary IL-1α, IL-6, IL-8 and TNF-α in patients with oral cancer were significantly higher than in patients with dysplastic oral lichen planus, inflammatory lesion that is often accompanied with epithelial discontinuity. Periodontal disease can also affect concentrations of salivary cytokines (27,28). However, no differences in periodontal health were ob-served between the groups in this study. 

Concentrations of salivary IL-1β and IL-6 in all three groups were not affected by smoking. Rhodus et al. (18) found no significant differences in concentrations of salivary cytokines between smokers and non smokers with oral cancer, oral lichen planus and healthy controls. Brailo et al. (29) found no differences in concentrations of salivary IL-6 and TNF-α between smokers and non smokers with oral leukoplakia and healthy controls. Results obtained in this study indicate that increased concentrations of salivary IL-1β and IL-6, rather than being caused by local inflammation, periodontal disease and smoking, reflect local production of these cytokines in cancer tissue. Oral cancer cells and tumor infiltrating lymphocytes are capable of producing all three studied cytokines (30). 

Based on the results of this study and previously mentioned roles of IL-1β and IL-6 in the process of malignant transformation, question arises whether these salivary cytokines could be used as markers of malignant transformation of oral leukoplakia. Increase in the production of proinflammatory cytokines is marked feature of cancerogenesis. Several studies report increased levels of proinflammatory cytokines in tissue or saliva of patients with oral cancer, which might support this assumption. Rhodus et al. (18) found increased concentrations of salivary IL-1α, IL-6, IL-8 and TNF-α in patients with oral cancer and oral lichen planus with epithelial dysplasia when compared to the healthy controls. Significant differences in concentrations of salivary IL-6 and IL-8 were also observed between patients with oral cancer and patients with oral lichen planus with epithelial dysplasia (18). 

Increase in the concentrations of proinflammatory cytokines in saliva might reflect the development of oral cancer from oral leukoplakia. Whether the increase of salivary IL-1β and IL-6 happens before oral cancer becomes clinically evident and whether it could be used for monitoring the malignant transformation of oral leukoplakia remains to be answered by further studies. Cross sectional study can not provide that answer but makes solid base for further research by identification and elimination of factors that might influence the values of studied cytokines. 
